# Qihuang needle therapy improves Tourette syndrome: A case report

**DOI:** 10.1097/MD.0000000000045844

**Published:** 2025-11-14

**Authors:** Yuyuan Tang, Xubeilei Liu, Zhirui Xu, Mengjiao Zhao, Lu Ding, Liming Lu, Chunzhi Tang, Bingxu Jin

**Affiliations:** a School of Chinese Medicine, Guangdong Jiangmen Chinese Medicine College, Jiangmen, China; b Department of Rehabilitation, Pan Yu Hospital Guangzhou University of Chinese Medicine, Guangzhou, China; c Clinical Research and Big Data Laboratory, South China Research Center for Acupuncture and Moxibustion, Medical College of Acu-Moxi and Rehabilitation, Guangzhou University of Chinese Medicine, Guangzhou, China.

**Keywords:** acupuncture, case report, Qihuang needle therapy, Tourette syndrome

## Abstract

**Rationale:**

Tourette syndrome (TS) is a chronic neuropsychiatric disorder characterized by motor and vocal tics, often associated with psychiatric comorbidities. It interferes with psychological development and quality of life. Although acupuncture has been reported as an effective treatment for tic disorders, its use in children is limited due to fear of needles and intolerance to needle retention. Qihuang needle (QH needle) therapy is a newly developed acupuncture modality with advantages of minimal pain, no need for needle retention, and rapid onset of effect. Here, we describe a pediatric case of TS successfully managed with QH needle therapy.

**Patient concerns:**

A 10-year-old boy presented with a 2-year history of recurrent multiple motor tics involving the face and limbs, along with vocal tics such as sniffing and throat clearing. The pharmacological treatment yielded suboptimal efficacy, with persistent symptom fluctuations prompting the consideration of complementary and alternative medicine.

**Diagnoses:**

The diagnosis of TS was established based on the patient’s medical history, the presence of multiple motor and vocal tics at some point in illness lasting more than 1 year, and age of onset at 8 years.

**Interventions:**

The patient was initially treated with pharmacological therapy to alleviate tic symptoms, however, the symptoms remained recurrent and poorly controlled. Repetitive transcranial magnetic stimulation was subsequently attempted for 10 consecutive days but showed limited efficacy and was discontinued due to poor compliance. Thereafter, QH needle therapy was administered over 14 sessions.

**Outcomes:**

Significant improvement was observed, with the Yale Global Tic Severity Scale score reduced from 52 to 14. No recurrence was reported during a 6-month follow-up period.

**Lessons:**

To our knowledge, this is the first reported case of TS treated with QH needle therapy. The results suggest that QH needle therapy may serve as a major or adjunctive treatment option for tic disorders and could be a promising method in pediatric disease management.

## 1. Introduction

Tourette syndrome (TS) is a neurodevelopmental disorders characterized by multiple motor and 1 or more vocal tics at some point in illness.^[1,2]^ Up to date, psychological intervention, behavioral therapy and pharmacological treatment are recommendations on treatment for TS. Nevertheless, the outcome of aforementioned treatments is unsatisfactory for various reasons.^[3]^ Patients under aged 9 years benefit less from behavioral therapy and its widespread use in China is hindered by a shortage of adequately trained professionals. Intolerance of side effects and low compliance reduce the acceptance of pharmacological treatment. Acupuncture shows good efficacy in TS.^[4]^ However, the limitations of traditional acupuncture restrict its application in pediatric patients.^[5]^ A child’s tolerance to the retention time of needle insertions is limited and their reluctance to remain still during traditional acupuncture treatments, which may expose them to safety risks and result in poor compliance. Auricular acupuncture perhaps is less pain but it requires children and their families to press to enhance its effectiveness. Most children with tic disorders are under academic pressure hence are difficult to complete treatment on time. An appropriate acupuncture method is needed for pediatric patients. The Qihuang (QH) needle therapy, a newly developed acupuncture modality, may be a better candidate due to its unique design and treatment rationale. Compared with conventional acupuncture modality, it features thicker needle and higher stiffness making it possible to effectively target affected meridian sinews, and delivering a stronger and sustained stimulus. Its acupoint selection, pattern differentiation, and acupuncture techniques are based on the traditional meridian sinew theory. Consequently, the QH needle therapy possesses unique advantages in the management of movement disorders including Parkinson’s disease (PD),^[6–9]^ essential tremor^[10]^, and progressive supranuclear palsy.^[10]^ In this case report, we aimed to present a case of a patient with TS, who experienced symptomatic relief after receiving treatment with the QH needle therapy.

## 2. Case presentation

On July 23, 2020, a 10-year-old male patient with a nearly 2-year history of tics, visited the Rehabilitation Department at Panyu Hospital of Chinese Medicine, presenting with tics characterized by frequent blinking, mouth twitching, sniffing and throat clearing. The patient’s tics began at the age of 8 without specific cause, initially presenting as eye blinking, and over time, tics gradually spread to trunk and lower extremities, manifesting as eye blinking, mouth twitching, involuntary leg lifting, and perineum tapping while walking. Vocal tics, including sniffing, throat clearing, and repetitive utterances of “ah huh” occurred following the motor tics. He had been diagnosed with tic disorder and treated with Tiapride hydrochloride, Inosine and Clonidine adhesive patch but his symptoms persisted with a waxing and waning course.

His medical history revealed that he was the first birth born, uneventfully and full term with a birth weight of 3.65 kg. The state of his birth was good, no cyanosis, apnea, convulsion, or bleeding. Typical child development milestones were met. The patient had a history of allergic rhinitis and eczema during early childhood. He had no history of dyskinesia induced by substances or medications nor any movement disorders cause by toxicity. He had no recent history of surgery or trauma and had received all scheduled vaccinations after birth.

No genetic diseases, including Down’s syndrome, Fragile X syndrome, tuberous sclerosis complex, neuroacanthocytosis, were reported. He had no family history of neurological or psychiatric diseases. At physical examination, his vital sign and neurological physical examination were normal. Laboratory tests showed no obvious abnormalities in blood trace elements, blood lead level, serum level of ceruloplasmin, rheumatoid factor, erythrocyte sedimentation rate, C-reactive protein, and antistreptolysin. A video electroencephalogram found no abnormal bioelectric signal.

## 3. Diagnosis

The diagnosis on arrival to our clinic was TS^[2]^ because he presented multiple motor and 1 or more vocal tics at some point in illness; his tics wax and wane, but have persisted more than 1 year since onset; the age of onset is before 18 years; and not caused by substance or other condition.

According to his medical history, laboratory test results, video electroencephalogram findings and negative physical examination, the following medical conditions and disorders were ruled out: infectious diseases including streptococcal infection, encephalitis, epileptic seizures, dystonia, myoclonus, and chorea. Secondary tic disorder was excluded because there was no specific cause and gradually worsening of tics, and no other neurologic signs or symptoms were simultaneously present.

## 4. Intervention

### 4.1. Pharmacological treatment (July 23, 2020 to November 29, 2020)

The patient was given western medicine, including tiapride (100 mg t.i.d), inosine tablets (10 mg b.i.d), vitamin B_6_ tablets (10 mg b.i.d) and vlonidine adhesive patches, along with the Chinese patent medicine Xiao’er Zhili Syrup (10 mL t.i.d). Tiapride and clonidine adhesive patch were used to alleviate tic symptoms. Inosine and vitamin B_6_ were provided to support neuronal health. Xiao’er Zhili Syrup was prescribed according to his traditional Chinese medicine (TCM) pattern.

### 4.2. Repetitive transcranial magnetic stimulation (September 27, 2020 to October 6, 2020)

**Figure 1.**
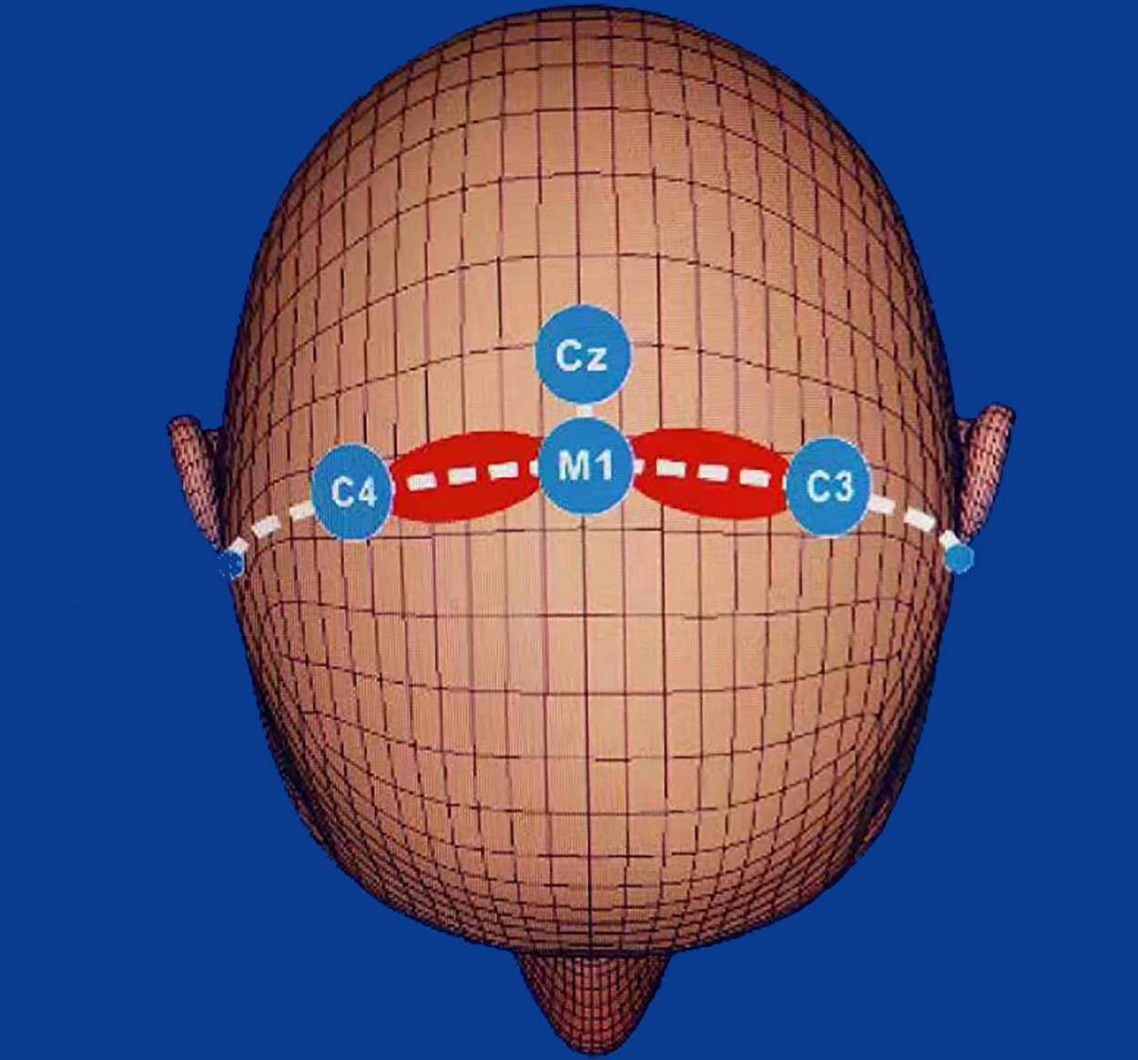
The stimulation positions of the rTMS. rTMS = repetitive transcranial magnetic stimulation.

After 1-month of discontinuing the medication, the patient’s tics gradually worsened. On the basis of previous treatment, a total of 10 sessions of low frequency (1 Hz) repetitive transcranial magnetic stimulation (rTMS) were administered over a period of 10 days. rTMS was administered at an intensity of 80% of the resting motor threshold. Each session consisted of 5 pulses followed by a 5 seconds rest, which was repeated 240 times (1200 stimuli/d), lasting a total of 39 minutes and 55 s/d. The stimulation position was left and right primary motor cortex (M1; Fig. 1).

### 4.3. Qihuang needle therapy (October 7, 2020 to December 27, 2020)

Acupuncture was performed by a certified acupuncturist with 20 years of clinical experience.

**Figure 2.**
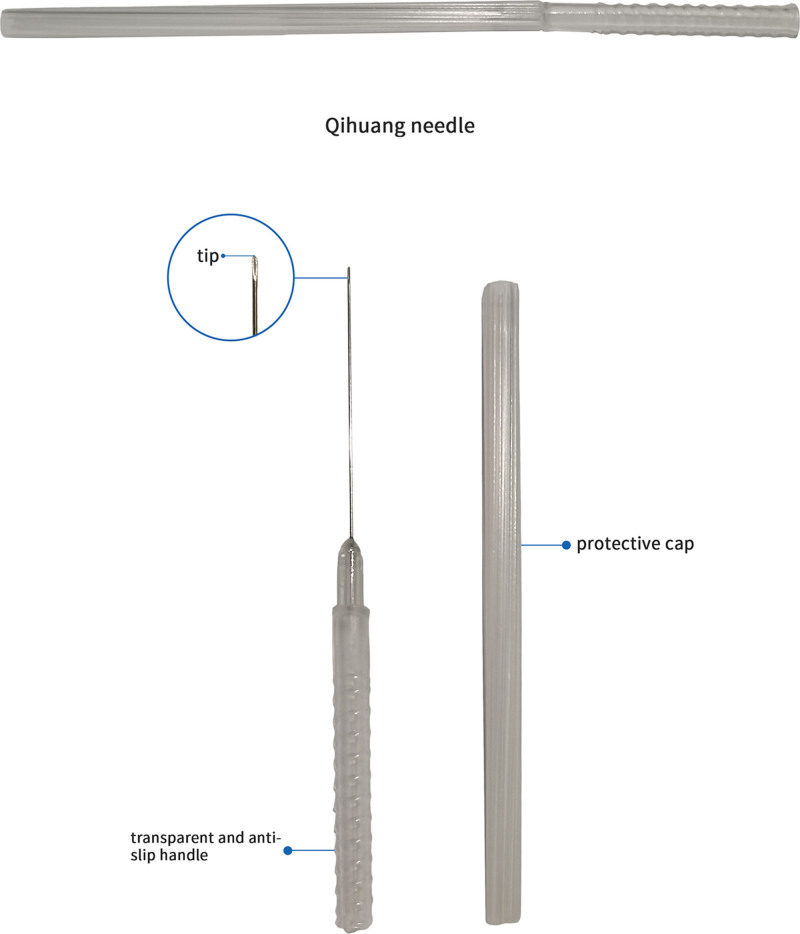
Qihuang needle.

**Figure 3.**
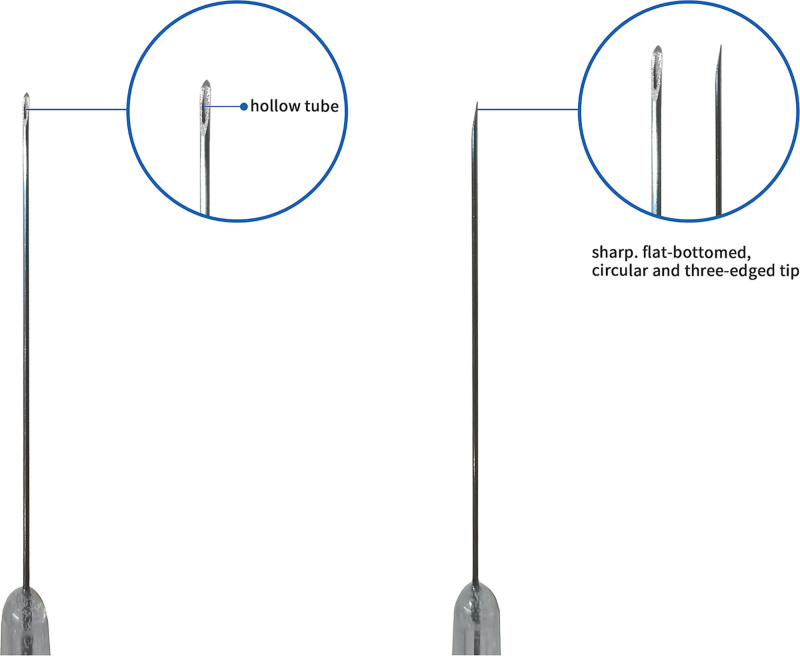
The hollow tube and sharp, arc-shaped tip of Qihuang needle.

QH needle is a newly developed acupuncture instrument invented by Dr Zhenhu Chen. Its design contributes to better therapeutic effects, reduced risk of adverse reactions, and improved patient compliance (Figs. 2 and 3). The design of QH needle draws inspiration from the long needle, the round-pointed needle, and the round-sharp needle described in the “Nine Classical Needles.”^[11]^ As for curative effect, the needle tip is sharp, flat-bottomed, circular and 3-edged, which facilitates smooth needle insertion, reduces puncture pain, and minimizes discomfort during manipulation, thus increasing patient acceptance and compliance and “Deqi” is pivotal for effective acupuncture. The needle body is a thick hollow tube, increasing stiffness, which enables stronger stimulus intensity and efficient transmission of “deqi” sensation along the needle body to the affected meridians and sinews. The increased stiffness also facilitates the precise depth insertion, allowing for various manipulations to induce qi. As for safety, the arc-shaped needle tip reduces the risk of hematoma, as contact with the vessel surface induces contraction of vascular smooth muscle, preventing direct penetration and the transparent and anti-slip handle facilitates the observation of bleeding in case the needle penetrates blood vessels repeatedly. In terms of compliance, the absence of needle retention shortens treatment duration, which was feasible for pediatric patients to receive treatment on time without negatively affecting their academic activities.

**Figure 4.**
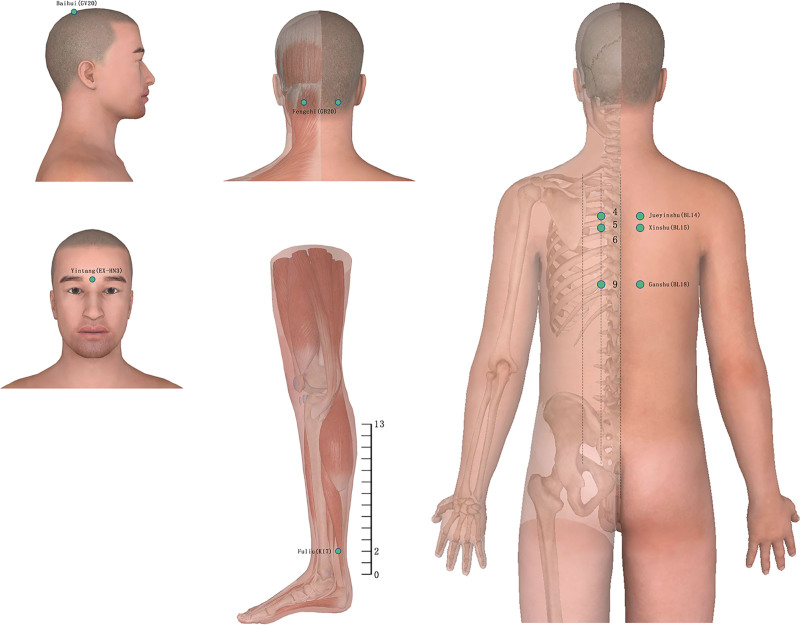
The selected acupoints.

Selected acupoints: Baihui (GV 20), Yintang (EX-HN 3), bilateral Jueyinshu (BL 14), bilateral Fengchi (GB 20) were selected for the first 2 weeks. Acupoints were adjusted subsequently because no further improvement was observed. Jueyinshu (BL14) was removed, and Xinshu (BL 15), Ganshu (BL 18) and Fuliu (KI 7) were added in the subsequent treatment (Fig. 4).

Course: QH needle therapy was administered twice a week for the first 2 weeks and once a week for the following treatment.

Manipulation: After skin disinfection, we pinched and lifted the flesh with a pressing hand. A QH needle (patent no. CN207412391U; 0.3 mm × 0.13 mm × 30 mm; QH; Chongqing) was inserted into the acupoints. For the GB 20 and KI 7, the needle was vertically inserted into the myofascial tissue. For the BL 14, BL 15, and BL 18, the needle was inserted obliquely along the spine until it reached the transverse process. For the GV 20, the needle was inserted perpendicularly into the galea aponeurotica then withdrawn slightly. For EX-HN 3, the needle was inserted obliquely into the subcutaneous tissue. After the “de qi” sensation was achieved (feelings of numbness, tingling, swelling, soreness, or muscle weakness), the handle of the needle was gently moved from side to side at an angle of 15° to 30° at a frequency of 80 to 120 times/min, for approximately 5 to 10 seconds. Once “de qi” sensation was obvious (usually after around 10 seconds of manipulation), the needle was withdrawn. The pinholes were then pressed with a sterile, dry cotton ball.

The patient was asked to select a comfortable posture for needling. The practitioner relaxed the patients and established a friendly and trusting environment before administering the acupuncture. Due attention is given to needling manipulation. Parents were asked to monitor their children carefully during acupuncture for emergency conditions. If any untoward incident occurred, appropriate measures were taken immediately.

## 5. Outcome and follow-up

### 5.1. The Yale Global Tic Severity Scale (YGTSS)

The YGTSS^[12]^ was used to measure the severity of tic symptoms. It is the most widely used rating scale for tic assessment both in clinical practice and research. It has the best evidence for assessing tic severity and indicates acceptable psychometric quality.^[13]^ An experienced trained pediatric clinician will carry out YGTSS by asking parents or guardians of participants to answer the items in YGTSS; observing participants’ performance on visits to the clinic. The total score (maximum rating of 100) is summing up the score of motor and vocal tics and functional impairment. YGTSS score < 25 indicates mild severity, between 25 and 50 suggests moderate and over 50 means severe. The change in the YGTSS score was detailed as follows.

1. Pharmacological treatment

In this stage, the patient’s YGTSS score decreased from 73 to 59. Motor tics were alleviated in terms of frequency, intensity, complexity and interference. Total motor tic severity score decreased from 25 to 21. He had discontinued Tiapride and Inosine since August 25th due to intolerance to side effects, including hypersomnia, dizziness, and gastrointestinal reactions. However, 2 weeks after discontinuing the medications, his eye blinking and perineum tapping gradually deteriorated. Vocal tics remained unchanged. The score of impairment reduced from 30 to 20.

1. Pharmacological treatment and 3 sessions of rTMS

There was minimal improvement in the total score of the YGTSS decreasing from 59 to 52.

1. Pharmacological treatment and 7 sessions of rTMS then pharmacological treatment and QH needle therapy

In this stage, the patient’s YGTSS decreased from 52 to 14. After completing 10 sessions of rTMS, the improvement in tic symptoms was not significant and rTMS was subsequently discontinued due to poor compliance. As a result, QH needle therapy was introduced. The parents reported that a partial alleviation of tics after the first QH needle therapy session. After 6 treatment sessions, the YGTSS score decreased to 38, indicating a reduction in tic severity from severe to moderate. Regarding motor tics, number and complexity decreased. Sequential facial tics were relieved, and leg lifting and perineum tapping were reduced. As for vocal tics, sniffing nearly disappeared, and the severity of interference from vocal tics decreased to a mild level.

The YGTSS score decreased from 38 to 14 over the next 4 treatment sessions, indicating a reduction in tic severity from moderate to mild. The score of total tic severity decreased from 28 to 14. Eye blinking and mouth twitching disappeared. Occasional perineum tapping and leg lifting were still observed, while throat clearing occurred only in the morning. His interactions with peers improved at school, and he gradually regained self-confidence during this period.

1. QH needle therapy

Motor tics and vocal tics resolved completely after receiving 4 single QH needle therapy.

**Figure 5.**
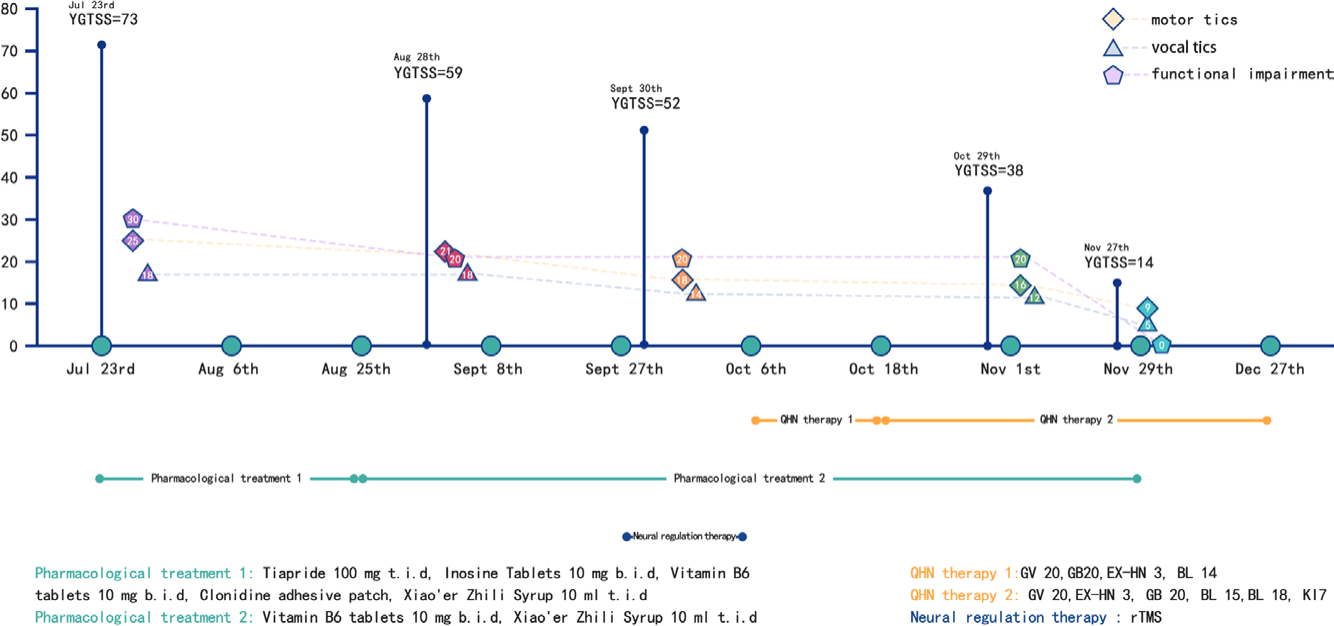
Treatment timeline.

No acupuncture-related adverse events were reported during the whole treatment period. Tics did not relapse during the 6-month follow-up. The process of treatment and changes in the YGTSS score are illustrated in Figure 5 and Table 1.

**Table 1:** Changes in severity scores on the YGTSS during treatment.

Measure	July 23, 2020		August 28, 2020		September 30, 2020		October 29, 2020		November 27, 2020	
Yale Global Tic Severity Scale	Motor tic	Vocal tic	Motor tic	Vocal tic	Motor tic	Vocal tic	Motor tic	Vocal tic	Motor tic	Vocal tic
Number	5	2	5	2	4	2	3	1	2	1
Frequency	5	4	4	4	3	3	3	3	2	1
Intensity	5	4	4	4	4	3	4	3	2	1
Complexity	5	4	4	4	4	3	3	3	2	1
Interference	5	4	4	4	3	3	3	2	1	1
Total tic severity score	25	18	21	18	18	14	16	12	9	9
Impairment	30		20		20		10		0	
Total YGTSS score	73		59		52		38		14	

The total tic severity score is the sum of the number, frequency, intensity, complexity and interference of tics, and ranges from 0 to 50. The total YGTSS includes scores for impairment and ranges from 0 to 100.

YGTSS = Yale Global Tic Severity Scale.

## 6. Discussion

Under the situation that the effect of conventional therapy is not satisfied in terms of effectiveness, safety or feasibility, acupuncture, as an easier and safer treatment method, has shown a positive effect on tic disorders. Acupuncture shows good efficacy, fewer adverse reactions, and a more flexible treatment cycle. Evidence indicated that its application in TS had good efficacy.^[14,15]^ However, the limitations of conventional acupuncture hinder its application. QH needle therapy could be a prospective method for the pediatric population.

Several clinical trials have investigated the effectiveness of QH needle therapy as an adjunctive treatment for patients with movement disorders. Its application in PD showed decreases in Unified Parkinson’s Disease Rating Scale Part III, NMSS, and Parkinson’s disease questionnaire-39 items compared with either filiform needle^[9]^ or sham acupuncture^[16]^ after 4 weeks of treatment. No rebound was reported after a 4- to 8-week follow-up, indicating its good long-term efficacy. Some case reports cover its effect on other movement disorders, including essential tremor, progressive supranuclear palsy^[10]^ and limb dysfunction after stroke.^[16]^ Its mechanism is perhaps related to its unique design, the treatment rationale derived from traditional meridian sinew theory, and the “five needling (method)” originated from the *Inner Canon of Huangdi*.^[17]^

The essential of meridian sinews is still in debate. It is proposed that meridian sinews have much in common with myofascial meridians.^[18]^ The holistic view of meridian sinews^[19]^ in TCM states that the meridian sinew classifies and arranges the sinews from the perspective of the lines of motion. It is an integrative structure with tensile linear soft tissues as the structure basis and the nervous system as the effect basis,^[20]^ so that the limbs and trunk form a regular 3-dimensional network structure framework, which are connected in structure and influence each other in function. Thus, the meridian sinew disease is related to disorders in the interactions between the central nervous system and peripheral mechanosensory responses.^[21]^

Acupuncture is a treatment that involves applying certain mechanical stimuli to the body. It is reported that needle insertion, manipulation, retention or pull-out could generate mechanical stimuli that may transmit mechanical signals into receptors and then induce biochemical and electrical signals.^[22]^ This process facilitates information communication throughout the body.

Needles and surrounding tissues develop multiple mechanical stimuli during needle manipulation. It is reported that lifting-thrusting, twirling, and waggling could generate mechanical stretch. Lifting-thrusting and twirling mainly increase friction. Waggling mainly increases pressure. Lifting-thrusting is the basic method in QH needling manipulation. Waggling and trembling are assisted needling methods. Evidence demonstrated that mechanical stretch stimuli at acupoints induce a faster and stronger signal compared to friction or pressure stimuli.^[23]^ The thicker, hollow QH needle body provides stronger mechanical stimuli to fascial connective tissue, and its needling methods increase mechanical stretch stimuli, for which QH needle yields a stronger sensation of “deqi” and sustained effects.

Thus, QH needle applies stronger mechanical stimuli at acupoints close to the affected meridian sinews. Local stimulation signals will be transmitted to the distant via meridian sinews with a faster conduction velocity than conventional acupuncture with the help of its design and manipulation methods. This may partly explain its better curative effect than conventional acupuncture.

This is the first report to our knowledge presenting a case of TS treated with QH needle therapy. The patient received various treatments, including pharmacological therapy (western medicine and TCM medication), as well as rTMS, but his tics symptoms remained fluctuating. Symptomatic relief was achieved after receiving QH needle therapy. As for the total tic severity score, QH needle therapy improved both motor tic (rating from 18 to 9) and vocal tic (rating from 14 to 5). The magnitude of the decrease in total tic score is greater after receiving QH needle therapy. The changes in each domain including number, frequency, intensity, complexity, and interference presented a greater decrease in both motor tics and vocal tics following QH needle therapy.

TS is categorized as a meridian sinew disorder in TCM. Its pathogenesis is that qi and blood fail to nourish meridian sinews, resulting in trembling of the tendons and muscles, tics, and contractures. Liver wind plays a central role in the pathogenesis, accompanied by involvement of 5 viscera. Meridian sinews treatments are aimed at the muscular system. According to the holistic view of the meridian sinew, the face, neck, upper back, and low back are connected through the bladder meridian sinew.^[19]^ In this case, the courses of the bladder meridian sinew and the gall bladder meridian sinew include muscles that involved in limbs and face tics. Thus, acupuncture at acupoints situated along these sinews induces widespread effects to alleviate tics. The brain (“sea of marrow”) governs the Shen (mind) and regulates movement, sensation, visceral functions, and mental activities. Its dysregulation impairs the meridian sinews, resulting in impaired motor control of the tendons and the development of tic disorders. The Governor vessel is the channel to the brain.^[24]^ Acupuncture at acupoints situated along the Governor vessel is thought to strengthen the functional connectivity between the peripheral and central nervous systems. Moreover, stimulation of the Back-Shu acupoints regulates the qi of the corresponding viscera and their functions. The afferent signals generated by needling these acupoints are transmitted to the brain via the spinal cord, inducing functional alterations that, in turn, enhance the interaction between the central and peripheral systems.^[25]^ Therefore, the selection of acupoints was primarily focused on these 3 meridian sinews. BL14, BL15, and BL18 are Back-Shu acupoints situated along the bladder meridian sinew, corresponding respectively to the Pericardium, Heart, and Liver. Specifically, stimulation of BL18 is traditionally believed to promote the flow of qi and blood along the Liver meridian, thereby regulating sinew function. GV20 and EX-HN3 are situated along the Governor vessel. Acupuncture at these acupoints is traditionally believed to regulate whole-body yang qi, as well as to warm and nourish the sinews.^[26]^ The combination of BL14, GV20 and EX-HN3 is to strengthen the connection between the heart and brain so as to regulating the Shen.^[27]^ GB20 is located along the gall bladder meridian sinew and is traditionally indicated for conditions attributed to pathogenic wind, particularly those involving neurological and musculoskeletal disturbances.^[28]^ The kidneys is believed, according to TCM theory, to have a deep connection to the brain through the spine, and cerebrospinal fluid and hormonal systems. It is proposed that sinews have anatomical, functional and theoretical affinity with the kidneys.^[29]^ Acupuncture at KI7 nourishes the kidney and replenishes Liver yin to tonify and relax sinews.

This case report is limited in a couple of ways. First, no objective index was measured, and currently, there are no recognized biological markers for the diagnosis or assessment of tic disorders. Inflammatory factors or neurotransmitters in plasma or serum are usually used to explore mechanisms. In addition, the pure effect of QH needle therapy could not be evaluated, as the patient presented with severe tics, and required comprehensive treatment. Nevertheless, QH needle therapy showed a positive effect in alleviating tic symptoms. As a single case report, the level of evidence is inherently limited. Future investigations employing rigorous study designs, objective outcome measures, and larger sample sizes are warranted to further evaluate the therapeutic efficacy of QH needle therapy and to elucidate its potential mechanisms.

This case showed the positive effect of QH needle therapy, whether used as a monotherapy or in combination with other treatments, in managing tic disorders. It is implicated that QH needle therapy could be used as a major or auxiliary treatment method for children diagnosed with tic disorders. Acupoints located along the affected meridian sinews and the Yang meridian sinews are frequently used. It is worth exploring the more specific clinical application of QH needle therapy in managing tic disorders in terms of its application among different types of tic disorders, optimal treatment sessions, standard of its manipulation, and so on.

## 7. Patient perspective

We were anxious because tics changed from 1 form to another, and new forms of tic emerged, although my child adhered to treatment. Tic had exerted a negative influence on his school performance and routine social life. We also worried about side effects of western medicine, but Chinese medication did not work. He had not received acupuncture before, and he was afraid of pain. It seems that tics showed minimal improvement after receiving pharmacological treatment and rTMS, and it is inconvenient for us to receive rTMS because of the academic activity. Our clinician proposed QH needle therapy, and we decided to give it a try. Treatment time became shorter, so we came to the clinic after class without distraction from his study. Symptoms were alleviated beyond our expectations after the first QH needle therapy, and my child showed more willingness to receive acupuncture. We were very pleased to see the remission.

## Author contributions

**Conceptualization:** Yuyuan Tang, Bingxu Jin.

**Funding acquisition:** Bingxu Jin, Liming Lu, Chunzhi Tang.

**Writing – original draft:** Yuyuan Tang, Xubeilei Liu.

**Writing – review & editing:** Yuyuan Tang, Zhirui Xu, Mengjiao Zhao, Lu Ding, Liming Lu.
